# Developing a Priority Scoring Index for Mobile Mammography Sites: Considerations for Screening Access in Rural and Remote Settings

**DOI:** 10.1177/1073274819883270

**Published:** 2019-11-01

**Authors:** Emma McKim Mitchell, Fabian Camacho

**Affiliations:** 1 Department of Family, Community & Mental Health Systems, University of Virginia School of Nursing, Charlottesville, VA, USA; 2 Department of Public Health Sciences, University of Virginia School of Medicine, Charlottesville, VA, USA

**Keywords:** mobile mammography, rural health, breast cancer screening, screening and early detection, Appalachian Virginia, Priority Index, vulnerable populations, cancer control

## Abstract

Geographic location continues to be an important indicator in incidence of, access to treatment for, and mortality from breast cancer. Disparities in access to screening and early detection persist in Appalachian Virginia. We developed an index to identify sites which would most benefit from increased frequency of mobile mammography visits, based on geographically relevant population-level risk factors (late stage of tumor diagnosis) and accessibility risk factors (access to FDA [US Food and Drug Administration] mammography sites, access of women aged 50+ years to primary care physicians at existing mobile sites). These 4 components for the Priority Index were subsequently standardized and multiplied to importance weights. The percentage of mammograms performed in the target geographic region has increased each year, respectively. This article presents methodological considerations for developing a priority algorithm to increase access to breast cancer early screening and detection for vulnerable women.

## Introduction

Geography remains an important predictor in breast cancer incidence, access to treatment,^1^ and mortality. Despite declining overall rates of cancer mortality, rural Appalachians continue to experience lower 3- and 5-year survival rates, as well as lower rates of early-stage diagnoses specifically for breast cancer, than either their non-Appalachian or nonrural counterparts.^2^ Receipt of regular primary care has been found to be an important indicator in increasing early detection of cancer in this region.^3^

Appalachian Virginia^4^ lies in the catchment area for the University of Virginia Health System and has been a strategic target area to develop innovative strategies to increase access to screening and early detection of breast cancer. For 15 years, the mobile mammography unit of the Health System has performed screening mammograms for women in this geographic region. In 2014, a new mobile coach was launched and has performed 5825 mobile mammograms across the Commonwealth of Virginia, with an average of 11% of these conducted in the tobacco footprint of far Southwest and Southside Virginia.^5^

### Purpose Statement

We aimed to develop an index to identify the mobile mammography sites which would most benefit from increased frequency of visits, based on geographically relevant population-level risk factors (late stage of tumor diagnosis) and accessibility risk factors (access to FDA [US Food and Drug Administration] mammography sites, access of women aged 50+ years to primary care physicians (PCP) at existing mobile sites). There is a continued need for evidence-based community-based outreach, and equally importantly, for stakeholder-informed priority setting to mitigate barriers and increase access to breast screening access. This article outlines a method other programs can use to facilitate prioritization of programmatic resources and offerings, particularly in rural and remote settings.

## Methods

### Priority Score Index for Mobile Mammography Sites

Four geographical and accessibility components were identified to quantify the benefit of increased frequency of screening visits: [a] Rate of late-stage tumor cancers in the area of the site, [b] spatial accessibility of women aged 50+ years to mobile mammography site, [c] spatial accessibility of all women to FDA facility mammography site, and [d] spatial accessibility of women to PCPs at mobile sites.

### Calculation of Advanced Stage Tumor Rates

County-level breast cancer tumor cases and advanced tumor cases were extracted from the Virginia Department of Health Cancer Registry from 2011 to 2015. Advanced tumor cases were identified using SEER stage variable (2 is regional by direct extension; 3 is regional by lymph nodes involved only; 4 regional by both direct extension and regional lymph node involvement; and 7 distant sites). In order to reduce sampling variation due to sparse sample sizes in some counties, the rates were smoothed by fitting a binomial regression with normally distributed random intercepts, accounting for spatial autocorrelation in the random effects by using a covariance matrix with spatial covariance matrix.^6^

### Calculation of Spatial Accessibility Indices

**Figure 1.**
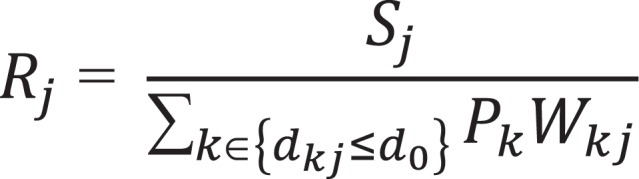
Formula used for index development.

The spatial accessibility index for components [b] to [d] was constructed based on an enhanced 2-step floating catchment area (2SFCA) method for measuring spatial accessibility^7,8^ using straight-line distances and population centroids calculated at the census tract level. The 2SFCA method has advantages over other traditional methods in measuring spatial accessibility.^9^ Provider to population ratios, one alternative, are confined to fixed boundaries and do not take into consideration patients crossing such boundaries, which may distort accessibility. Travel time and distance measures, another alternative, do not take into account supply and demand factors that affect health care.^10,11^ Furthermore, the 2SFCA has been shown to have empirical validity in predicting clinical outcomes.^12,13^ In the first step of this analysis, a supply to population ratio was measured using the following formulas for each component (Equation 1):

a. Mobile mammography site *j* supply to surrounding 50+ population:

where *S_j_* was set to 1 representing a supply of 1 site per location. The sum in the denominator captures surrounding population and was conducted over all county centroids *k* within a distance *d*_0_ from location *j* ($d_{kj}$)*;*P_k_** represents the population of women aged 50+ years at county *k* (2010 Census Estimates), and $W_{kj}$ was a linearly decaying weight as a function of $d_{kj}$ and reaching 0 at *d*_0_. The cutoff distance *d*_0_ was set at a default of *50 miles from each location.* There were a total of 154 mammography sites. County population estimates were not restricted to Virginia but incorporated surrounding states if necessary.

b. FDA mammography facility site *j* supply to surrounding population:

The same formula (Figure 1) was used, with *S_j_* set to 1 and $P_{k\;}$ set to the female county population. FDA mammography sites in Virginia and surrounding states were identified from the FDA-provided sites during the latest available year (2018), for a total of 2228 facilities.

c. PCP site *j* supply to the surrounding population:

The same formula (Figure 1) was used, with *j* representing each county PCP supply located at county *j* centroid.$\;S_{j}\;$ was set to the number of PCP at county *j*, where PCP was evaluated as the number of office-based general practitioners, family medicine, and general internists in 2015, as provided by the Area Health Resource File from the Virginia Department of Health and Human Services. *P_k_* was set to the female county population.

In the second step, the surrounding supply ratios at mobile mammography site *i* were summed to produce the spatial accessibility index $A_{i}^{F}$ according to the formula:

$$A_{i}^{F}=\underset{j\in \left\{d_{ij}\leq d_{0}\right\}}{\sum }R_{j}W_{ij}$$

where *R_j_* represented the supply ratio at point *j* within a distance $d_{0\;}$ of site *i* and $W_{ij}$ was a linearly decaying weight as a function of distance $d_{ij}\;$ from point *j* to site *i*, reaching 0 at *d*_0_. As in A, *d*_0_ was set to a distance of 50 miles. $A_{i}^{F}$ is a measure of the accessibility at site *i* to mobile mammography sites (including *i*), accessibility to surrounding FDA mammography sites, and accessibility to surrounding PCP.

### Calculation of Priority Index

The 4 components for the Priority Index were standardized and multiplied to importance weights as follows: 4 points to standardized advanced rates, 3 points to standardized mobile mammography accessibility, 2 points to standardized FDA mammography accessibility, and 1 point for standardized PCP accessibility. The weighted components were then sum-scored. Accessibility was reverse-scored so that higher priority could coincide with lower accessibility.

## Results

**Figure 2.**
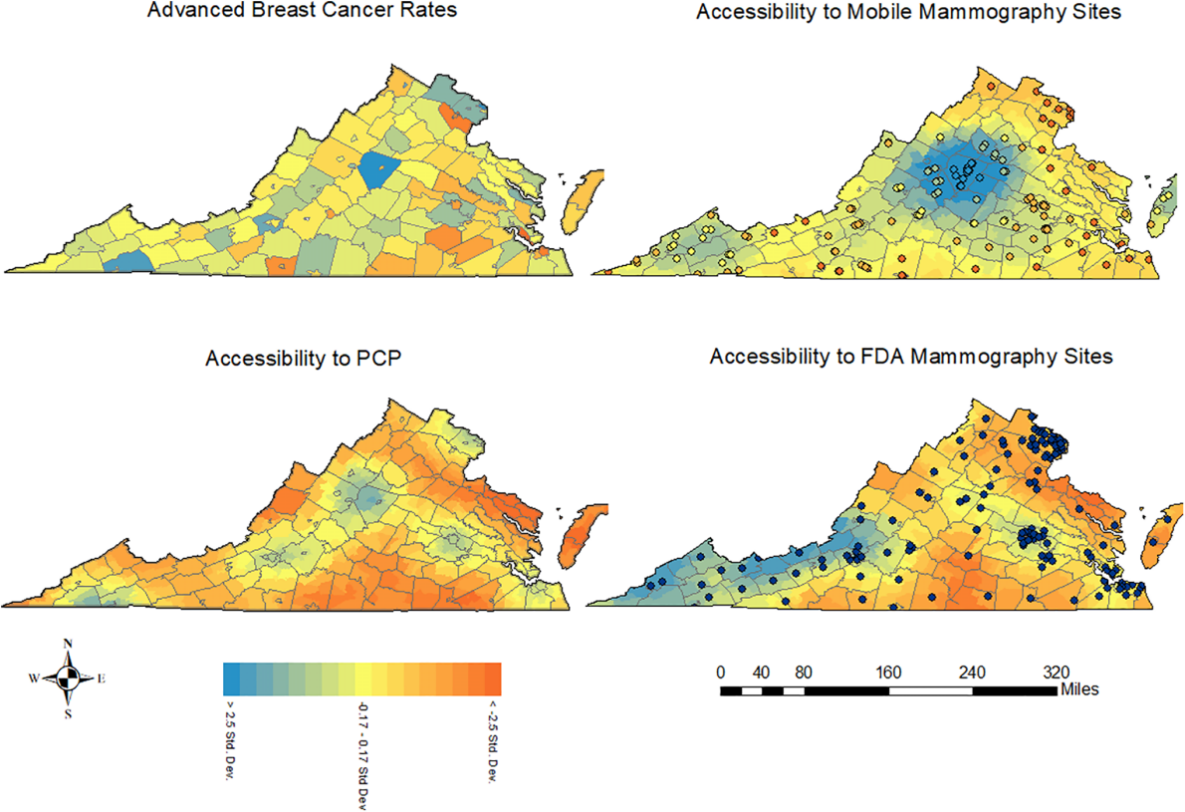
Cloropleth maps for individual components of priority score. *Note*. Scores are standardized with mean = 0 and standard deviation of 1. Bluer regions coincide with desirable outcomes, including lower advanced breast cancer rates and improved accessibility. Mobile mammography and FDA mammography sites’ points are shown in corresponding maps. Advanced rates are shown at the county level, while accessibility is shown for each census tract in the state. The maps were created using a 50-mile cutoff.

Figure 2 displays the scores for the individual components of the index throughout the state, where advanced cancer rates are displayed at the county level and accessibility index was evaluated for each census tract. The scores were standardized to have a mean of zero and a standard deviation of 1. Bluer regions coincide with desirable outcomes such as lower advanced breast cancer rates as well as improved accessibility. Dots in the mobile and FDA maps show geolocation of mobile mammography and FDA mammography sites.

We infer that regions of relative PCP accessibility are observed in local regions centered around more urban regions of Virginia, including Charlottesville, Northern Virginia, Richmond, Norfolk, and Washington County bordering the Tennessee Tri Cities region. Regions of particular rates of advanced cancer coincide with counties surrounding Charlottesville, Roanoke, and the Tri-Cities region. Mobile mammography accessibility is particularly high in a circular region around Charlottesville; FDA accessibility was found to be higher in western Virginia.

**Figure 3:**
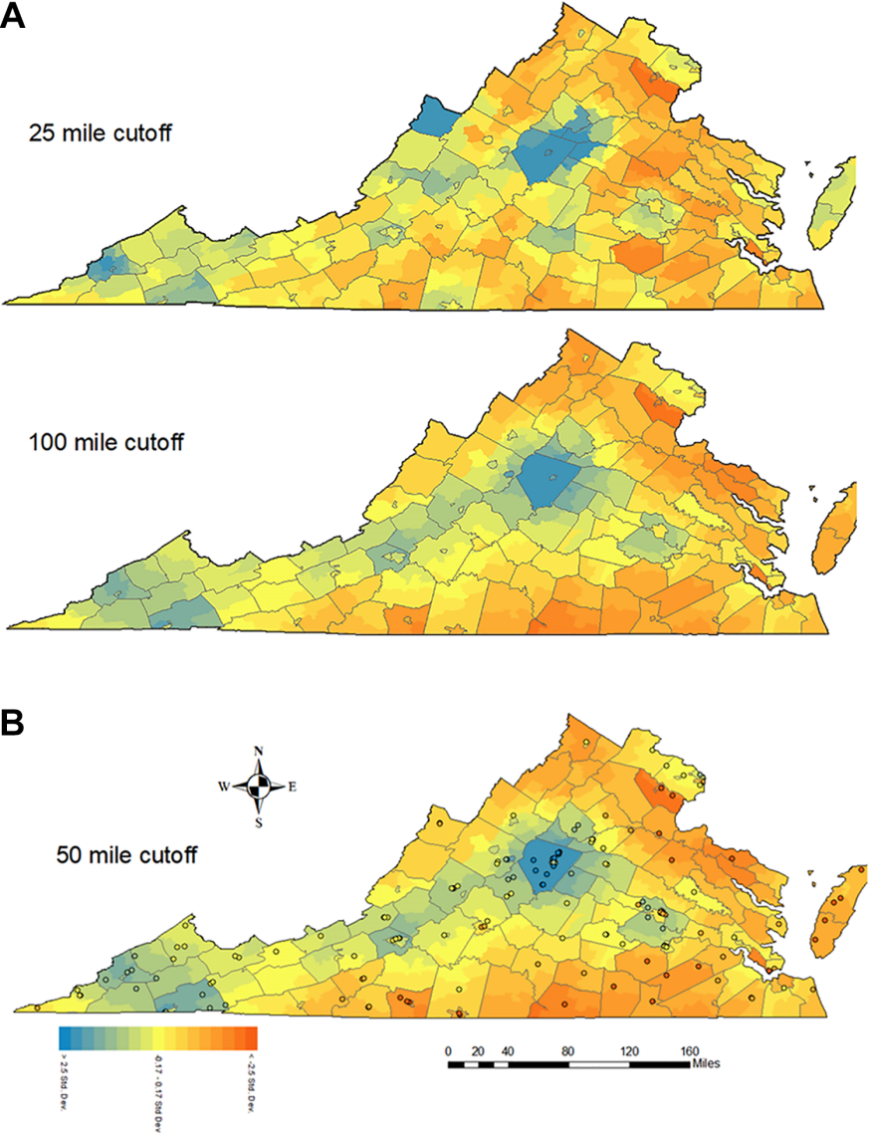
A and B, Composite priority score map by cutoff. *Note*. Scores are standardized with 0 mean and 1 standard deviation.

Figure 3A displays the composite priority score with the original cutoff of 50 miles (*d*_0_). Regions needing high priority coincide with south-central Virginia and a regional band in northeastern Virginia. Conversely, the region centering around Charlottesville as well as Southwest Virginia scores lower in priority, suggesting higher accessibility and lower relative advanced cancer rates in this region. We additionally investigated how scores depend on the cutoff used (*d*_0_) by changing the cutoff to 25 and 100 miles instead of 50 (Figure 3B). A smaller cutoff distance results in accessibility with more emphasis on more local facilities, PCP, and sites, while ignoring contributions from more distant sites. The intra-class correlation coefficient, a measure of inter-rater reliability, among these 3 scores is estimated at 83%, suggesting the ratings are robust against variation of the thresholds within the examined range.

### Screening Outcomes

Using the formulas outlined above, an index was designed to inform the prioritization algorithm detailed herein. This algorithm was first created in the summer of 2014. The percentage of mammograms performed in the target geographic region has increased each year, respectively: in 2014, 1165 mammograms were completed with 4% in the target region versus in 2016 when 1746 mammograms in total were completed with 14.2% within the target geographic region.

## Discussion

There are several parameters that were chosen in designing the algorithm which could be modified, including the priority components, the weighting of the components, and the use of straight-line distances, thresholds, and decay function when calculating 2-stage accessibility index. Although for this presentation indices are calculated over all the state, the application of the index required calculating it at each mobile mammography site. As a next step, then, the sites were ranked from high to low and the top sites were selected as requiring most needed based on the index.

Choice of the parameters could potentially result in different priority rankings.

Regarding the use of straight-line distances, we are of the view that although driving distance may be preferable for added precision, given the geography in the study region and the high correlations between both measures in the most of US geography,^14^ the choice may largely be inconsequential for nonemergency travels.^14^ In addition, the convenience of straight-line distances allows for more rapid deployment and updating as of the present decade.

Allowing for decay addresses objections in the literature that accessibility within a catchment area is not necessarily equal.^7^ However, there may be a subjective element in the choice of decay function, which has been most frequently assumed as linear. We sought to address impact of these variations in decay by conducting sensitivity analysis that varied the threshold *d_0_*, resulting in different rates of decay.

Regarding the choice of components, they were developed after a review of the literature, in consultation with the University of Virginia Cancer Center Without Walls Community Advisory Board and in consultation with clinicians and researchers. We think, however, that weighting of the components could be made more objective and view our current more “subjective” assignments as a potential limitation. An objective approach, for example, could be to conduct a discrete choice experiment (DCE),^14,15^ which would experimentally elicit the importance stakeholders attach to each component. The standard DCE method would result in a survey where the stakeholders choose between pairs of scenarios of attribute combinations. The number of scenarios to ask (choice sets) would be based on a fractional factorial design^16^ to avoid excessive response burden and cost. Component weights could then be derived by analyzing the responses to the survey using DCE methods. This approach may be of particular relevance to similar mobile mammography programs seeking to adapt this methodology to practice context and region-specific considerations and priorities.

## Conclusions

This article presents methodological considerations for developing a priority algorithm to increase access to breast cancer early screening and detection for vulnerable women. There is a continued need for evidence-based community-based outreach, and equally importantly, for stakeholder-informed priority setting to mitigate barriers and increase access to breast screening access. This article outlines a method other programs can use to facilitate prioritization of programmatic resources and offerings, particularly in rural and remote settings.

## Supplemental Material

Supplemental Material, completed_SQUIRE_checklist_12_13_18_Cancer_Control - Developing a Priority Scoring Index for Mobile Mammography Sites: Considerations for Screening Access in Rural and Remote Settings Click here for additional data file. Supplemental Material, completed_SQUIRE_checklist_12_13_18_Cancer_Control for Developing a Priority Scoring Index for Mobile Mammography Sites: Considerations for Screening Access in Rural and Remote Settings by Emma McKim Mitchell and Fabian Camacho in Cancer Control
